# Perceived Community Belonging as a Moderator of the Association Between Sexual Orientation and Health and Well-Being

**DOI:** 10.1177/08901171231204472

**Published:** 2023-10-03

**Authors:** Lei Chai

**Affiliations:** 1 Department of Sociology, 7938 University of Toronto, Toronto, ON, Canada

**Keywords:** sexual orientation, health, well-being, community belonging, Canada

## Abstract

**Purpose:**

This study examines the moderating role of perceived community belonging in the association between sexual orientation and various health and well-being outcomes.

**Design:**

A national cross-sectional survey.

**Setting:**

Confidential microdata from the 2021 Canadian Community Health Survey.

**Subjects:**

Individuals aged 15 and older, with a sample size ranging from 43,000 to 44,100.

**Measures:**

Sexual orientation, health and well-being outcomes, and sense of community belonging were all self-reported. Outcomes included self-rated general and mental health, depressive symptoms, and life satisfaction.

**Analysis:**

A series of multiple linear regression models.

**Results:**

Compared to heterosexual individuals, bisexual individuals reported poorer self-rated general health (b = .402, *P* < .001 for men; b = .454, *P* < .001 for women) and mental health (b = .520, *P* < .001 for men; b = .643, *P* < .001 for women), higher depressive symptoms (b = 2.140, *P* < .001 for men; b = 2.685, *P* < .001 for women), and lower life satisfaction (b = .383, *P* < .05 for men; b = .842, *P* < .001 for women). Few disparities were observed among gay men and lesbians. Contrary to some recent findings, no disparities were observed among individuals uncertain about their sexual orientation or those who chose not to disclose it, even without controlling for covariates. A stronger sense of community belonging mitigated the disadvantages associated with self-rated general health (b = -.276, *P* < .01) and depressive symptoms (b = -.983, *P* < .01) for gay men, and life satisfaction (b = -.621, *P* < .01) for lesbians.

**Conclusion:**

This study is among the first to highlight the stress-buffering role of community belonging in the association between sexual orientation and health and well-being outcomes.

A vast body of research has highlighted the impact of sexual orientation on various health and well-being outcomes.^1-7^ Using the minority stress model as a guiding framework, scholars suggest that sexual minority individuals often experience heightened health disparities compared to heterosexuals, owing to increased exposure to minority stress.^8,9^ This stress can manifest in forms of internalized homophobia, victimization, and discrimination, all of which are rooted in individuals’ stigmatized sexual identities.^8,9^ Numerous studies have confirmed that sexual minority individuals face more pronounced physical and mental health challenges, with bisexual individuals showing greater disparities than gays and lesbians.^10-14^ These patterns are also evident in subjective well-being measures, such as life satisfaction, and in health-related outcomes like sleep problems and substance use.^11,15-17^

The health and well-being disadvantages faced by bisexuals may stem from negative stereotypes surrounding bisexuality, which differ from those associated with homosexuality.^18-20^ Existing research has indicated a societal inclination to treat bisexuality as a ‘choice,’ primarily because bisexuals express romantic interests in both genders.^19,21^ Reinforcing this perspective, a study by Eliason^22^ found that only 26% of respondents agreed with the statement that “people are probably born bisexual.” In contrast, there is a growing societal consensus acknowledging that gay men and lesbians are likely born with their sexual orientation.^23^ This differential stance, based on perceived ‘choice,’ contributes to societal views that often depict bisexuals as selfish, immature, and less competent compared to their heterosexual, gay, and lesbian counterparts.^24^ This perspective is consistent with the broader observation that stigmatized traits perceived as controllable frequently encounter heightened discrimination.^25^ If society perceives certain characteristics as controllable, those possessing them may face increased blame for their marginalized status.^19^ In the context of bisexuality, these perceptions may suggest that society views bisexuals as responsible for their orientation, thereby possibly justifying discrimination against them.^19^

While a wealth of research exists on this topic, the present study seeks to advance prior work by addressing three critical limitations. First, much of the existing research has been US-centric, raising questions about the applicability of such findings in other countries. This investigation delves into the association between sexual orientation and health and well-being outcomes in Canada. With its comparatively more accepting attitude towards sexual diversity than the US,^26^ Canada offers a compelling setting for this exploration. This progressive attitude was evident when Canada legalized same-sex marriage in 2005, a full decade before the US.^27^ Furthermore, Canada’s public health care system could potentially mitigate some of the health disparities linked to sexual orientation.^28,29^ However, as the system primarily focuses on physical health and hospital-related care,^30^ significant mental health disparities may still exist among sexual minority individuals.^31^

Existing Canadian studies that explore the association between sexual orientation and health present varied results. Evidence indicates that both homosexual and bisexual individuals are more likely to report mood and anxiety disorders than heterosexuals.^32^ Some studies have stratified their analysis by gender, showing that sexual minority men and women are more likely to report mood disorders than their heterosexual counterparts, respectively.^33^ A few investigations focus exclusively on one gender. Such studies have indicated that gay and bisexual men do not show significant differences in self-rated general and mental health, smoking, and drinking outcomes relative to heterosexual men.^34^ In contrast, bisexual women are more likely to report worse self-rated general and mental health compared to heterosexual women, while differences between lesbian and heterosexual women are minimal.^35^

These varied results emphasize the need for a more in-depth examination. This study seeks to build on prior Canadian investigations by distinguishing between homosexual and bisexual individuals and stratifying the analysis by gender. Employing recent data from the 2021 Canadian Community Health Survey (CCHS)—the only national survey incorporating a sexual orientation variable with a range of health and well-being measures—this study considers self-rated mental and general health, life satisfaction, and, following Statistics Canada’s 2021 initiative, depressive symptoms. Based on the minority stress model, it is hypothesized that gay, lesbian, and bisexual individuals will report poorer self-rated mental and general health, higher depressive symptoms, and lower life satisfaction compared to heterosexual individuals (Hypothesis 1). Furthermore, it is anticipated that bisexual individuals will report greater disparities compared to homosexual individuals (Hypothesis 2).

Second, while there is significant interest in examining the association between sexual orientation and health and well-being outcomes, few studies have specifically addressed individuals who do not respond to questions about sexual orientation. Recent literature reveals a portion of respondents, comparable to those identifying as gay men or lesbians, who report non-responses like “don’t know” or “refusal.”^36,37^ Notably, this pattern of non-response is not random; older participants or those with a lower educational background are less likely to report their sexual orientation, potentially due to a lack of understanding of the question.^38^ Furthermore, individuals from certain cultural backgrounds (eg, Chinese, South Asian, Southeast Asian, or Arab) are also less likely to answer a question on sexual orientation,^39^ possibly due to stigmas associated with their sexual minority status.^40^

Despite the potential health disadvantages these individuals may face, most research has not viewed them as a distinct sexual orientation subgroup in a multivariate framework. The only Canadian study addressing this issue, conducted by Sivakumaran and Margolis,^40^ found that middle-aged and older adults reporting “don’t know” in response to the sexual orientation question were more likely to report poor self-rated general and mental health relative to their heterosexual counterparts. However, some of these disparities were fully accounted for by covariates. To the best of the author’s knowledge, no study has estimated this association in the broader general population. Nevertheless, the study by Sivakumaran and Margolis^40^ provides some evidence to support the hypothesis that individuals reporting “don’t know” or “refusal” to the sexual orientation question will experience poorer self-rated mental and general health, higher depressive symptoms, and lower life satisfaction compared to their heterosexual counterparts (Hypothesis 3).

Third, the current understanding of potential coping resources that may alleviate health and well-being disparities among sexual minority individuals is limited. This oversight is significant, particularly considering the disproportionate experiences of chronic stress reported by sexual minority individuals. The stress process model^41^ posits that stressors originating from a disadvantaged social status can influence an individual’s health and well-being. However, these effects may be buffered or moderated by coping resources, such as social support. Extensive research has revealed the protective role of social support in mitigating adverse life events and chronic strains among sexual minority individuals.^42-44^ Yet, given the potentially limited access to social support resulting from family and peer rejection,^32^ there is a growing call among health scholars for the identification of potential community-level coping resources.^45^

Community belonging, a type of geographically-based social capital, is defined as “the connectedness, attachment, and commitment a person makes to social relationships in the community.”^46(p1394)^ Studies have shown that community belonging can act as a proxy indicator for social support^47^ and thereby enhance various health outcomes.^47-51^ Furthermore, multiple studies have confirmed the stress-buffering role of community belonging among immigrants and racial/ethnic minority populations.^52-56^ Applied to the present scenario, a strong sense of community connectedness could provide a less stigmatized living environment for sexual minority individuals, facilitating more positive identity development.^32^ Moreover, a strong sense of community belonging may bolster self-esteem, promote social engagement and participation, and foster health-enhancing behaviors such as a balanced diet and regular physical activity among sexual minority individuals.^45,57^

Unfortunately, few studies have explored its moderating potential in the association between sexual orientation and health and well-being. A notable exception is a study based on the 2007-2012 CCHS by Pakula and colleagues.^32^ Contrary to expectations, they did not find evidence supporting the stress-buffering role of community belonging. Instead, they discovered that the penalties associated with mood disorders were *amplified* among homosexual respondents (ie, combining gays and lesbians) with a stronger sense of community belonging. Despite this unexpected finding, drawing from research about social support and community belonging, it is plausible that a stronger sense of community belonging will alleviate disadvantages associated with self-rated mental and general health, depressive symptoms, and life satisfaction among gay, lesbian, and bisexual individuals (Hypothesis 4).

## Methods

### Data

To test the proposed hypotheses, this study utilized data from the confidential microdata file of the 2021 Canadian Community Health Survey (CCHS).^58^ The CCHS is a nationally representative cross-sectional survey administered by Statistics Canada. It collects information on health status, health determinants, and health care utilization patterns of Canadians aged 12 and older. However, the survey excludes residents of Indian reserves, health care institutions, some remote areas, and full-time members of the Canadian Forces. Data collection was conducted through both telephone and in-person interviews. Participation in the survey was voluntary, and informed consent was obtained from all participants. See Statistics Canada for further details about the CCHS sample design and data collection procedures.^58^

Since the question concerning sexual orientation was directed only at individuals aged 15 and older, 1,793 respondents between the ages of 12 and 14 were excluded from the analysis. Missing values for the selected variables were minimal, ranging between .14 and 3.74%. This made listwise deletion an appropriate procedure for handling them. The final sample size for analyzing self-rated mental and general health, as well as life satisfaction, was 44,100. However, due to missing data concerning depressive symptoms in 1,100 individuals, the final sample size for analyzing depressive symptoms was reduced to 43,000.

### Measures

Self-rated mental health was measured with the question: “In general, would you say your mental health is…?” Responses were coded as “excellent” (1), “very good” (2), “good” (3), “fair” (4), and “poor” (5). This measure was treated as a continuous variable.^59,60^

Self-rated general health was measured with the question: “In general, would you say your health is…?” Responses included “excellent” (1), “very good” (2), “good” (3), “fair” (4), and “poor” (5). This measure was also treated as a continuous variable.^59,61^

Life satisfaction was measured with the question: “Using a scale of 0 to 10, where 0 means ‘very dissatisfied’ and 10 means ‘very satisfied,’ how do you feel about your life as a whole right now?” This item was then reverse-coded.

Depressive symptoms were measured with nine established items from the Patient Health Questionnaire (PHQ-9).^62^ Respondents were asked to indicate how often over the previous two weeks they had experienced a range of problems: “little interest or pleasure in doing things,” “feeling down, depressed, or hopeless,” “trouble falling or staying asleep, or sleeping too much,” “feeling tired or having little energy,” “poor appetite or overeating,” “feeling bad about yourself— or that you are a failure or have let yourself or your family down,” “trouble concentrating on things, such as reading the newspaper or watching television,” “moving or speaking so slowly that other people could have noticed? Or the opposite—being so fidgety or restless that you have been moving around a lot more than usual,” and “thoughts that you would be better off dead, or of hurting yourself in some way.” Each item was coded as “not at all,” “several days,” “more than half the days,” and “nearly every day.” A scale was created by summing the responses, ranging from 0 to 27, with higher scores indicating increased depressive symptoms (alpha = .81). However, due to high skewness, responses were top-coded at 10.^63,64^

Sexual orientation was identified through self-reporting with the question: “What is your sexual orientation?” Responses were coded as “heterosexual,” “gay or lesbian,” “bisexual or pansexual,” “not elsewhere classified,” “don’t know,” “refusal,” and “not stated.” Respondents who reported “not elsewhere classified” and “not stated” were excluded from the analysis due to small sample sizes.

Gender was coded as “men,” “women,” and “gender diverse.” Respondents identifying as “gender diverse” were excluded from the analysis due to a small sample size.

Local community belonging was measured with the question: “How would you describe your sense of belonging to your local community? Would you say it is…?” Responses were coded as “very weak” (1), “somewhat weak” (2), “somewhat strong” (3), and “very strong” (4). This measure was treated as a continuous variable.^52,65^

The study controlled for several socio-demographic variables that could potentially confound the association between sexual orientation and health and well-being. *Age* was coded in years. *Race/ethnicity* was coded as “White,” “non-White,” and “Aboriginal.” *Marital status* was coded as “married,” “cohabiting,” “previously married,” and “single/never married.” *Household size* was coded as a continuous variable. *Education* was coded as “less than secondary school graduation,” “secondary school graduation, no post-secondary education,” and “post-secondary certificate, diploma, or university degree.” *Main activity* was coded as “working or on vacation (from paid work),” “looking for paid work,” “going to school (including on vacation from school),” “retired,” “long-term illness,” and “other.” *Total household income* was coded as “no income or income loss,” “less than $5,000,” “$5000 to $9,999,” “$10,000 to $14,999,” “$15,000 to $19,999,” “$20,000 to $29,999,” “$30,000 to $39,999,” “$40,000 to $49,999,” “$50,000 to $59,999,” “$60,000 to $69,999,” “$70,000 to $79,999,” “$80,000 to $89,999,” “$90,000 to $99,999,” “$100,000 to $149,999,” and “$150,000 or more.” *Province* was coded as “Newfoundland and Labrador,” “Prince Edward Island,” “Nova Scotia,” “New Brunswick,” “Quebec,” “Ontario,” “Manitoba,” “Saskatchewan,” “Alberta,” and “British Columbia.” Table 1 presents the descriptive statistics for all study variables.

**Table 1.** Descriptive Statistics of Selected Variables in the Study.

	Full Sample	Heterosexual Men	Gay Men	Bisexual Men	Don’t know Men	Refusal Men	Heterosexual Women	Lesbian Women	Bisexual Women	Don’t know Women	Refusal Women
	Mean or %	Mean or %	Mean or %	Mean or %	Mean or %	Mean or %	Mean or %	Mean or %	Mean or %	Mean or %	Mean or %
Self-rated general health	2.31 (.01)	2.28 (.01)	2.38 (.10)	2.64 (.13)	2.38 (.21)	2.23 (.16)	2.31 (.01)	2.26 (.10)	2.67 (.06)	2.65 (.15)	2.48 (.12)
Self-rated mental health	2.31 (.01)	2.20 (.01)	2.49 (.11)	2.91 (.15)	2.48 (.16)	2.24 (.18)	2.37 (.01)	2.42 (.12)	3.29 (.07)	2.58 (.13)	2.57 (.15)
Life satisfaction	2.06 (.01)	2.03 (.02)	2.24 (.16)	2.63 (.20)	1.88 (.38)	2.20 (.30)	2.05 (.02)	2.03 (.18)	3.03 (.10)	2.30 (.17)	2.36 (.23)
Depressive symptoms	2.87 (.03)	2.40 (.04)	3.43 (.31)	5.45 (.48)	1.54 (.56)	1.84 (.43)	3.11 (.04)	3.74 (.34)	6.99 (.24)	3.36 (.48)	3.73 (.46)
Community belonging	2.79 (.01)	2.78 (.01)	2.64 (.09)	2.52 (.11)	3.04 (.17)	2.97 (.08)	2.80 (.01)	2.70 (.08)	2.53 (.06)	2.81 (.11)	2.56 (.13)
Age	47.36 (.17)	46.91 (.26)	43.69 (1.62)	30.96 (1.45)	40.77 (3.72)	47.58 (3.70)	48.95 (.23)	41.27 (1.63)	27.90 (.84)	51.05 (3.47)	50.21 (1.84)
Race/ethnicity											
White	73.19	72.45	67.9	71.12	Removed	64.39	73.9	82.33	77.67	Removed	71.26
Non-white/Aboriginal	26.81	27.55	32.1	28.88		35.61	26.1	17.67	22.33		28.74
Marital status											
Married	48.18	51.01	17.21	15.95	41.49	44.5	48.45	20.26	13.70	28.18	50.77
Unmarried	51.82	48.99	82.79	84.05	58.51	55.5	51.55	79.74	86.30	71.82	49.23
Household size	2.80 (.01)	2.84 (.02)	2.18 (.15)	3.32 (.18)	3.55 (.33)	2.87 (.28)	2.76 (.02)	2.53 (.12)	3.23 (.09)	2.23 (.13)	2.50 (.17)
Education											
Less than secondary school	11.30	11.75	4.61	15.16	39.26	12.80	10.50	12.96	15.42	28.51	16.74
Secondary school graduation	21.93	22.32	14.37	23.60	36.53	32.73	21.37	17.05	28.44	22.09	22.46
Post-secondary certificate diploma	66.77	65.93	81.02	61.23	24.21	54.47	68.13	69.98	56.14	49.41	60.80
Main activity											
Working	53.31	60.03	58.28	43.36	34.72	55.83	47.26	57.85	44.75	30.01	42.37
Other	46.69	39.97	41.72	56.64	65.28	42.17	52.74	42.15	55.25	69.99	57.63
Household income											
Low	36.80	33.44	41.50	35.12	32.32	47.02	39.63	42.48	36.25	61.15	55.11
High	63.20	65.56	58.50	64.88	67.68	52.98	60.37	57.52	63.75	38.85	44.89
Province											
Ontario	39.09	39.12	35.09	36.82	70.60	63.84	39.11	28.99	34.30	47.56	43.31
Other	60.91	60.88	64.91	63.18	29.40	36.16	60.89	71.01	65.70	52.44	56.69
N	44100	18800	350	200	100	150	23280	250	550	200	200
Percentage	100	42.63	.79	.45	.23	.34	52.79	.57	1.25	.45	.45

Note: Weighted means or percentages (%s) are presented, but ns are unweighted. Standard errors are in parentheses. Following the vetting rules of Statistics Canada, race/ethnicity, marital status, main activity, household income (with the median category as the cut-off point), and province are dummy-coded to ensure that the cell sizes are large enough across sexual orientation subgroups. The unweighted ns are also rounded. Four cells are removed due to small sample sizes.

### Analytical Strategy

The present study employed a series of Ordinary Least Squares (OLS) regression models to test the proposed hypotheses. All models included a full set of control variables and weights provided by the survey. The analysis began with Models 1-4 in Table 2, which explored the association between sexual orientation and four outcomes—self-rated mental health, self-rated general health, life satisfaction, and depressive symptoms. These models were conducted separately for men (Panel A) and women (Panel B) to account for potential gender-specific differences.^12^ Additionally, Table 3 tested the interaction terms. Models 1-4 examined whether the association between sexual orientation and each health and well-being outcome varied across levels of community belonging. These models were also stratified by gender, with men represented in Panel A and women in Panel B. All analyses were performed using Stata/SE 14.2.

**Table 2.** Ordinary Least Squares Regression Predicting Self-Rated Mental and General Health, Life Satisfaction, and Depressive Symptoms.

	Model 1	Model 2	Model 3	Model 4
	SR mental health	SR general health	Life satisfaction	Depressive symptoms
	b	b	b	b
Panel A: Men				
Sexual orientation (SO)				
Heterosexual (ref)				
Gay	.211 (.112)	.118 (.099)	.029 (.169)	.670* (.328)
Bisexual	.520*** (.147)	.402*** (.124)	.383* (.194)	2.140*** (.437)
Don’t know	.206 (.139)	.059 (.184)	-.186 (.303)	-1.174** (.400)
Refusal	-.017 (.181)	-.118 (.140)	.020 (.306)	-.671 (.448)
Intercept	2.724	2.389	2.335	3.726
N	19,600	19,600	19,600	19,100
Panel B: Women				
Sexual orientation (SO)				
Heterosexual (ref)				
Lesbian	-.061 (.097)	.006 (.099)	-.111 (.172)	.187 (.301)
Bisexual	.643*** (.076)	.454*** (.060)	.842*** (.098)	2.685*** (.249)
Don’t know	.163 (.133)	.214 (.133)	.094 (.170)	.090 (.496)
Refusal	.177 (.145)	.120 (.121)	.234 (.236)	.560 (.442)
Intercept	3.099	2.354	2.714	5.991
N	24,500	24,500	24,500	23,900

Note: “SR” = self-rated and “ref” = reference. All models include a full set of control variables: Age, race/ethnicity, marital status, household size, education, main activity, household income, and province. All models are weighted. Following the vetting rules of Statistics Canada, the sample sizes are rounded. ***P < .001; **P < .01; *P < .05.

**Table 3.** Ordinary Least Squares Regression Predicting Self-Rated Mental and General Health, Life Satisfaction, and Depressive Symptoms, Moderation by Community Belonging.

	Model 1	Model 2	Model 3	Model 4
	SR mental health	SR general health	Life satisfaction	Depressive symptoms
	b	b	b	b
Panel A: Men				
Sexual orientation (SO)				
Heterosexual (ref)				
Gay	.762* (.302)	.833*** (.252)	.883* (.447)	3.205*** (.883)
Bisexual	.908** (.330)	.327 (.355)	-.111 (.595)	3.221** (1.094)
Don’t know	.387 (.474)	.750 (.495)	.995 (.844)	.116 (1.406)
Refusal	.376 (.449)	-.382 (.443)	1.319 (.804)	.473 (1.371)
Community belonging (CB)	-.288*** (.017)	-.234*** (.017)	-.538*** (.028)	-.815*** (.053)
SO × CB				
Gay × CB	-.214 (.116)	-.276** (.093)	-.336 (.172)	-.983** (.317)
Bisexual × CB	-.176 (.138)	.012 (.132)	.172 (.219)	-.484 (.396)
Don’t know × CB	-.042 (.153)	-.212 (.152)	-.355 (.261)	-.365 (.406)
Refusal × CB	-.117 (.147)	.101 (.142)	-.410 (.264)	-.331 (.407)
Intercept	3.406	2.945	3.566	5.677
N	19,600	19,600	19,600	19,100
Panel B: Women				
Sexual orientation (SO)				
Heterosexual (ref)				
Lesbian	.314 (.339)	.520 (.332)	1.533* (.719)	1.287 (.799)
Bisexual	.807*** (.195)	.449** (.164)	.869* (.348)	2.561*** (.661)
Don’t know	.326 (.515)	.397 (.524)	.535 (.662)	-2.529 (1.796)
Refusal	.260 (.396)	.330 (.334)	.059 (.711)	-.229 (1.210)
Community belonging (CB)	-.270*** (.015)	-.216*** (.014)	-.504*** (.025)	-.831*** (.050)
SO × CB				
Lesbian × CB	-.145 (.110)	-.199 (.113)	-.621** (.234)	-.421 (.277)
Bisexual × CB	-.087 (.070)	-.015 (.061)	-.051 (.129)	-.023 (.268)
Don’t know × CB	-.057 (.161)	-.064 (.157)	-.154 (.215)	.937 (.544)
Refusal × CB	-.059 (.127)	-.103 (.113)	.019 (.215)	.228 (.388)
Intercept	3.764	2.884	3.948	8.066
N	24,500	24,500	24,500	23,900

Note: “SR” = self-rated and “ref” = reference. All models include a full set of control variables: Age, race/ethnicity, marital status, household size, education, main activity, household income, and province. All models are weighted. Following the vetting rules of Statistics Canada, the sample sizes are rounded. ***P < .001; **P < .01; *P < .05

## Results

Table 2 presents OLS regression models predicting self-rated mental and general health, life satisfaction, and depressive symptoms, stratified by gender. Models 1-4 of Panel A demonstrated that bisexual men reported poorer self-rated mental (b = .520, *P* < .001) and general (b = .402, *P* < .001) health, lower life satisfaction (b = .383, *P* < .05), and higher depressive symptoms (b = 2.140, *P* < .001) compared to heterosexual men, with few disparities among gay men and those who reported “don’t know” or “refusal” to the sexual orientation question.

Similar patterns were observed for women: Models 1-4 of Panel B revealed that bisexual women reported poorer self-rated mental (b = .643, *P* < .001) and general (b = .454, *P* < .001) health, lower life satisfaction (b = .842, *P* < .001), and higher depressive symptoms (b = 2.685, *P* < .001) compared to heterosexual women. However, no disparities were observed among lesbians and those reporting “don’t know” or “refusal” to the sexual orientation question. Collectively, these findings partially supported Hypothesis 1 that gay, lesbian, and bisexual individuals would report poorer self-rated mental and general health, higher depressive symptoms, and lower life satisfaction, compared to heterosexual individuals; fully supported Hypothesis 2 that bisexual individuals would report greater disparities compared to homosexual individuals; and failed to support Hypothesis 3 that individuals who reported “don’t know” or “refusal” to the sexual orientation question would experience poorer self-rated mental and general health, higher depressive symptoms, and lower life satisfaction compared to their heterosexual counterparts.

**Figure 1.**
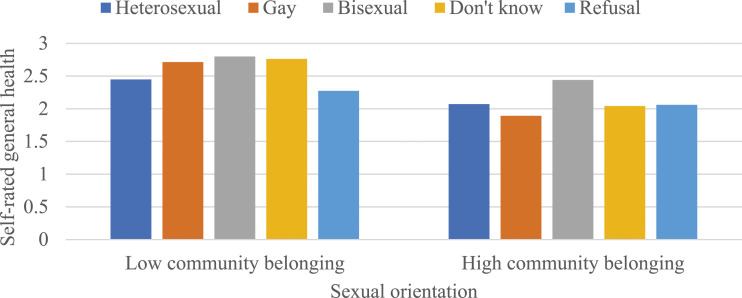
The moderating effects of community belonging on the association between sexual orientation and self-rated general health among men. Note: Results are based on Model 2 of Panel A in Table 3. Low and high community belonging represent -1 and +1 standard deviations from the mean, respectively.

**Figure 2.**
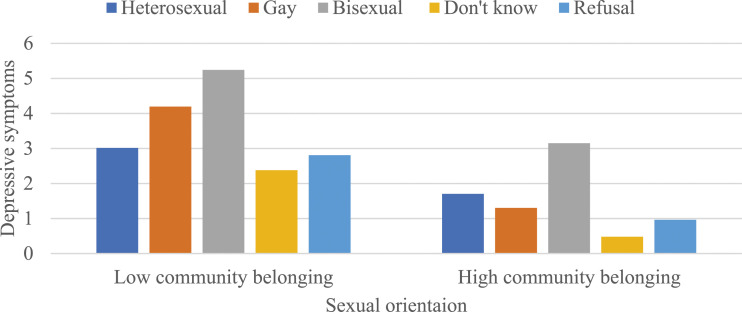
The moderating effects of community belonging on the association between sexual orientation and depressive symptoms among men. Note: Results are based on Model 4 of Panel A in Table 3. Low and high community belonging represent -1 and +1 standard deviations from the mean, respectively.

Table 3 presents OLS regression models predicting the moderating effects of community belonging on the association between sexual orientation and each health and well-being outcome, stratified by gender. While community belonging did not moderate the associations between sexual orientation and self-rated mental health and life satisfaction among men (as shown in Models 1 and 3 of Panel A), there were negative and statistically significant interactions between sexual orientation and community belonging in predicting self-rated general health (b = -.276, *P* < .01) and depressive symptoms (b = -.983, *P* < .01) (as shown in Models 2 and 4 of Panel A). To interpret these 2 coefficients, predicted values were calculated (see Figures 1 and 2). These values indicated that a stronger sense of community belonging attenuated the disadvantages in self-rated general health and depressive symptoms among gay men.

**Figure 3.**
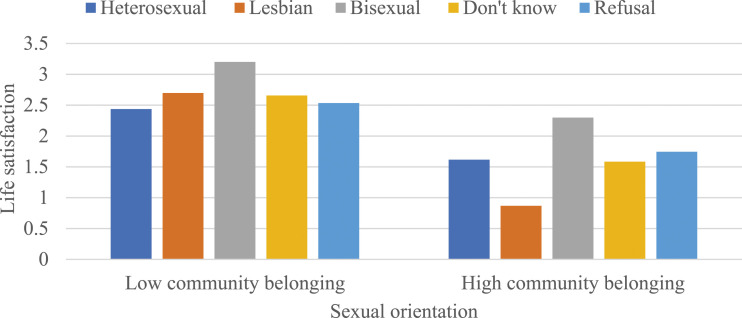
The moderating effects of community belonging on the association between sexual orientation and life satisfaction among women. Note: Results are based on Model 3 of Panel B in Table 3. Low and high community belonging represent -1 and +1 standard deviations from the mean, respectively.

Somewhat different patterns were observed among women in Panel B; that is, community belonging did not moderate the associations between sexual orientation and self-rated mental and general health and depressive symptoms (as shown in Models 1, 2, and 4), but there was a negative and statistically significant interaction between sexual orientation and community belonging in predicting life satisfaction (b = -.621, *P* < .01). To interpret this coefficient, predicted values were calculated once again (see Figure 3). These values indicated that a stronger sense of community belonging attenuated the disadvantages in life satisfaction among lesbians. Collectively, these findings partially supported Hypothesis 4 that a stronger sense of community belonging would alleviate disadvantages associated with self-rated mental and general health, depressive symptoms, and life satisfaction among gay, lesbian, and bisexual individuals.

## Discussion and Conclusion

Drawing from the most recent cycle of the CCHS, this study examined the association between sexual orientation and multiple health and well-being outcomes, considering the moderating influence of perceived community belonging. Several findings emerged from this investigation. First, consistent with prior research in the United States,^10-14^ it was observed that bisexual men and women reported poorer self-rated mental and general health, lower life satisfaction, and higher depressive symptoms compared to their heterosexual counterparts. This supports the minority stress perspective, suggesting that due to their stigmatized sexual identity, sexual minority individuals experience more adverse health and well-being outcomes.^9^ Interestingly, few disparities were observed among gay men and lesbians.

Although this result aligns with the notion that bisexual individuals face greater health and well-being disparities compared to homosexual individuals due to negative stereotypes associated with bisexuality,^20,24,25^ it contradicts findings from the United States, where there is a general consensus that gay men and lesbians report worse health outcomes compared to heterosexuals.^10-14^ For instance, Liu and Reczek,^13^ utilizing the 2013-2018 National Health Interview Survey, highlighted more significant disparities in self-rated general health among gays and lesbians. The lack of significant disparities observed in this study may be attributed to Canada’s more supportive attitudes toward sexual diversity and the protective influence of the publicly funded health care system,^26,28,29^ underscoring the importance of taking the social context into account when researching the association between sexual orientation and health and well-being.

Some readers may question if the grouping of sexual orientation subgroups could affect the observed patterns. To address this, several additional models were conducted. The patterns from Appendix A showed that when gay men and lesbians were combined, the results were largely consistent with those presented in Table 2. However, according to Appendix B, when homosexual and bisexual individuals were grouped together, sexual minority men and women reported worse health and well-being outcomes, with the exception of life satisfaction among sexual minority men. This suggests that bisexual individuals, a subgroup at high risk of health and well-being disparities, could drive the association for the non-heterosexual category. Future research in Canada should thus avoid lumping homosexual with bisexual respondents into a single group, when documenting health and well-being consequences to ensure more accurate information.

Second, in response to recent appeals for more research to investigate potential health and well-being consequences among respondents who do not disclose their sexual orientation,^40^ this study revealed that those who reported “don’t know” or “refusal” to the sexual orientation question experienced no disparities across all four outcomes. One plausible explanation could be the relatively small sample size of those who did not report their sexual orientation. To test this claim, the models from Table 2, combining “don’t know” with “refusal,” were conducted. This approach increased the proportion of nonresponse, making it comparable to that of sexual minority individuals. However, the results remained statistically insignificant (see Appendix C).

Another potential explanation is that the penalties among those who reported nonresponse were fully explained by covariates such as age, race/ethnicity, and education.^40^ To evaluate this claim, the models from Table 2, without controlling for any covariates, were conducted (see Appendix D). The results remained largely insignificant, except for self-rated general health among lesbians, which does not support the claim that “low socioeconomic status, rather than discrimination and stigma, is driving poor health among those who did not know their sexual orientation.”^40(p1755)^ It is important to note, though, that in their study, Sivakumaran and Margolis^40^ did not identify a consistent pattern since the penalties among those not responding to the sexual orientation question varied across age groups and health outcomes. Consequently, many questions remain unanswered about the health consequences among this group of individuals, highlighting the need for further research given their non-negligible size.

Third and possibly one of the most novel findings of this study, pertains to the moderating effects of community belonging. It was found that a stronger sense of community belonging alleviated the disparities in self-rated general health and depressive symptoms among gay men, and life satisfaction among lesbians. These findings align with the stress-buffering role that community belonging plays in mitigating stressful experiences among socioeconomically disadvantaged groups like immigrants and racial/ethnic minorities.^52-56^

However, these results also raise questions as to why the moderating effects of community belonging varied based on specific outcomes. Only one Canadian study, to the best of the author’s knowledge, has evaluated the moderating potential of community belonging among sexual minority individuals: Pakula and colleagues^32^ found that community belonging *intensified* the disparities in mood disorders but not anxiety disorders, among homosexual individuals. They speculated that “while a strong sense of local community belonging may offer a ‘safe place’ and serve as a buffer that reduces minority stress for some individuals, for others, established local community norms and behavior might be associated with negative outcomes, including pronounced awareness of one’s social disadvantage.”^32(p1189-1190)^ Future research utilizing a mixed-method approach could offer more insightful perspectives into the moderating role of community belonging among sexual minority individuals.

This study has its limitations. First, there is increasing recognition among scholars regarding the importance of documenting health disparities by sexual orientation from an intersectional perspective.^66-68^ Despite using nationally representative data from the 2021 CCHS, the cell sizes were inadequate to estimate the relationship between sexual orientation and health and well-being across various socio-demographic groups. Some researchers have attempted to overcome this limitation by pooling data from multiple waves and combining gay/lesbian individuals with bisexual individuals.^68^ However, their findings may still be somewhat conservative. Second, sexual orientation is a multidimensional construct that can be measured by sexual identity, sexual attraction, or sexual behavior.^19^ Unfortunately, measures of sexual attraction and sexual behavior were not available in the CCHS, except for the 2015-2016 cycle. Emerging research has begun to explore how discordance in sexual orientation can shape health and well-being outcomes.^69^ Thus, future studies should consider multiple dimensions of sexual orientation when estimating health and well-being consequences.

Despite these limitations, the present study offers the most recent and comprehensive analysis of health and well-being disparities by sexual orientation and gender using the 2021 CCHS data. Furthermore, this study is among the first to examine and confirm the stress-buffering role of perceived community belonging. These findings can inform the development of public health interventions aimed at improving the health and well-being of sexual minority individuals.

## Appendix A. OLS regression predicting self-rated mental and general health, life satisfaction, and depressive symptoms.

**Table:**

	Model 1	Model 2	Model 3	Model 4
	SR mental health	SR general health	Life satisfaction	Depressive symptoms
	b	b	b	b
Sexual orientation (SO)				
Heterosexual (ref)				
Gay/lesbian	.120 (.081)	.077 (.073)	-.009 (.123)	.523* (.233)
Bisexual	.634*** (.069)	.448*** (.055)	.722*** (.090)	2.627*** (.217)
Don’t know	.166 (.097)	.145 (.111)	-.029 (.165)	-.453 (.333)
Refusal	.083 (.119)	.002 (.097)	.134 (.193)	-.005 (.334)
Intercept	2.832	2.381	2.531	4.496
N	44,100	44,100	44,100	43,000

Note: All models included a full set of control variables: gender, age, race/ethnicity, marital status, household size, education, main activity, household income, and province. All models were weighted. Following the vetting rules of Statistics Canada, the sample sizes were rounded. ****P* < .001; *p<. 05.

## Appendix B. OLS regression predicting self-rated mental and general health, life satisfaction, and depressive symptoms.

**Table:**

	Model 1	Model 2	Model 3	Model 4
	SR mental health	SR general health	Life satisfaction	Depressive symptoms
	b	b	b	b
Panel A: Men				
Sexual orientation (SO)				
Heterosexual (ref)				
Gay/bisexual	.328*** (.091)	.226** (.080)	.163 (.130)	1.226*** (.276)
Don’t know	.203 (.139)	.056 (.184)	-.190 (.303)	-1.189** (.398)
Refusal	-.017 (.181)	-.118 (.140)	.019 (.307)	-.672 (.449)
Intercept	2.725	2.390	2.336	3.727
N	19,600	19,600	19,600	19,100
Panel B: Women				
Sexual orientation (SO)				
Heterosexual (ref)				
Lesbian/bisexual	.460*** (.068)	.338*** (.054)	.594*** (.092)	2.042*** (.222)
Don’t know	.163 (.133)	.214 (.133)	.094 (.170)	.090 (.496)
Refusal	.177 (.145)	.120 (.121)	.234 (.236)	.550 (.441)
Intercept	3.111	2.362	2.730	6.043
N	24,500	24,500	24,500	23,900

Note: All models included a full set of control variables: age, race/ethnicity, marital status, household size, education, main activity, household income, and province. All models were weighted. Following the vetting rules of Statistics Canada, the sample sizes were rounded. ****P* < .001; ***P* < .01.

## Appendix C. OLS regression predicting self-rated mental and general health, life satisfaction, and depressive symptoms.

**Table:**

	Model 1	Model 2	Model 3	Model 4
	SR mental health	SR general health	Life satisfaction	Depressive symptoms
	b	b	b	b
Panel A: Men				
Sexual orientation (SO)				
Heterosexual (ref)				
Gay	.211 (.112)	.118 (.099)	.029 (.169)	.670* (.328)
Bisexual	.520*** (.147)	.402*** (.124)	.383* (.194)	2.140*** (.437)
Don’t know/refusal	.062 (.130)	-.055 (.112)	-.053 (.224)	-.855** (.317)
Intercept	2.724	2.389	2.335	3.726
N	19,600	19,600	19,600	19,100
Panel B: Women				
Sexual orientation (SO)				
Heterosexual (ref)				
Lesbian	-.061 (.097)	.006 (.099)	-.111 (.172)	.187 (.301)
Bisexual	.643*** (.076)	.454*** (.060)	.842*** (.098)	2.685*** (.249)
Don’t know/refusal	.171 (.101)	.161 (.090)	.173 (.154)	.361 (.339)
Intercept	3.099	2.354	2.714	5.991
N	24,500	24,500	24,500	23,900

Note: All models included a full set of control variables: age, race/ethnicity, marital status, household size, education, main activity, household income, and province. All models were weighted. Following the vetting rules of Statistics Canada, the sample sizes were rounded. ****P* < .001; ***P* < .01; **P* < .05.

## Appendix D. OLS regression predicting self-rated mental and general health, life satisfaction, and depressive symptoms, without covariates.

**Table:**

	Model 1a	Model 1b	Model 1c	Model 1d
	SR mental health	SR general health	Life satisfaction	Depressive symptoms
	b	b	b	b
Panel A: Men				
Sexual orientation (SO)				
Heterosexual (ref)				
Gay	.286** (.109)	.104 (.102)	.210 (.162)	1.027*** (.309)
Bisexual	.706*** (.154)	.358** (.129)	.605** (.206)	3.053*** (.482)
Don’t know	.278 (.165)	.107 (.212)	-.149 (.374)	-.861 (.557)
Refusal	.044 (.180)	-.045 (.161)	.172 (.299)	-.558 (.428)
Intercept	2.200	2.277	2.026	2.400
N	19,600	19,600	19,600	19,100
Panel A: Women				
Sexual orientation (SO)				
Heterosexual (ref)				
Lesbian	.055 (.115)	-.051 (.105)	-.018 (.185)	.635 (.342)
Bisexual	.926*** (.033)	.358*** (.062)	.982*** (.097)	3.877*** (.247)
Don’t know	.212 (.132)	.340* (.146)	.250 (.171)	.249 (.484)
Refusal	.204 (.147)	.174 (.123)	.312 (.235)	.622 (.457)
Intercept	2.368	2.310	2.049	3.108
N	24,500	24,500	24,500	23,900

All models were weighted. Following the vetting rules of Statistics Canada, the sample sizes were rounded. ****P* < .001; ***P* < .01.

SO WHAT? What is already known on this topic? Extensive research provides compelling evidence that sexual minority individuals report heightened physical and mental health problems, with bisexual individuals showing even more substantial disparities than gay and lesbian individuals. What does this article add? The current research addresses three significant limitations. First, most studies focus on the United States, raising questions about the applicability of these disparities to other countries. Second, few studies have specifically addressed individuals who do not respond to questions about sexual orientation. Third, the current understanding of potential coping resources that could mitigate these disparities remains limited. What are the implications for health promotion practice or research? Few disparities were observed among gay men and lesbians in Canada, underscoring the importance of taking the social context into account. No disparities were found among individuals who were uncertain about their sexual orientation or chose not to disclose it. The significant number of such individuals highlights the need for further research. A stronger sense of community belonging alleviated the disadvantages associated with self-rated general health and depressive symptoms for gay men, and life satisfaction for lesbians. This information can inform the development of public health interventions aimed at improving the health and well-being of sexual minority individuals.
